# Quantitative evaluation of range and metabolic activity of hepatic alveolar echinococcosis lesion microenvironment using PET/CT and multi-site sampling method

**DOI:** 10.1186/s12879-021-06366-3

**Published:** 2021-07-23

**Authors:** Abudusalamu Aini, Maiweilidan Yimingjiang, Aimaiti Yasen, Bo Ran, Tiemin Jiang, Xiaohong Li, Jian Wang, Abuduaini Abulizi, Zhide Li, Yingmei Shao, Tuerganaili Aji, Hao Wen

**Affiliations:** 1 Hepatobiliary and Echinococcosis Surgery Department, First Affiliated Hospital of Xinjiang Medical University, Urumqi, China; 2 Xinjiang Uyghur Autonomous Region Clinical Research Center for Echinococcosis and Hepatobiliary Diseases, First Affiliated Hospital of Xinjiang Medical University, Urumqi, China; 3 State Key Laboratory of Pathogenesis, Prevention and Management of High Incidence Diseases in Central Asia, Xinjiang Medical University, Urumqi, China; 4 Department of Pathology, First Affiliated Hospital of Xinjiang Medical University, Urumqi, China; 5 Digestive and Vascular Surgery Center, First Affiliated Hospital of Xinjiang Medical University, Urumqi, China; 6 Department of Nuclear Medicine, First Affiliated Hospital of Xinjiang Medical University, Urumqi, China; 7 Radiology Center, First Affiliated Hospital of Xinjiang Medical University, Urumqi, China; 8 WHO Collaboration Center on Prevention and Management of Echinococcosis, Clinical Medical Institute, First Affiliated Hospital of Xinjiang Medical University, Urumqi, China

**Keywords:** Alveolar echinococcosis (AE), Multi-site sampling (MSS), PET/CT, Lesion microenvironment (LME), Immune infiltration

## Abstract

**Background:**

Alveolar echinococcosis (AE) lesion microenvironment (LME) is crucial site where parasite-host interactions happen and of great significance during surgery and obtaining liver samples for basic research. However, little is known about quantification of LME range and its’ metabolic activity regarding different lesion characteristics.

**Methods:**

A prospective and retrospective analysis of LME from surgical AE patients was performed. Patients (*n* = 75) received abdominal computed tomography (CT) and position emission tomography/computed tomography using ^18^F-fluodeoxyglucose (^18^F-FDG-PET/CT) within 1 week prior to surgery. Semiquantitatively, calcification was clustered with 0%, < 50% and ≥ 50% degrees at lesion periphery; liquefaction was clustered with 0%, < 50%, 50 ~ 75%, ≥75% degrees at lesion center using volumetric ratio. Tumor to background ratio (TBR) of ^18^F-FDG standard uptake value (SUV, *n* = 75) was calculated, and range of ^18^F-FDG uptake area was measured; Multi-site sampling method (MSS, *n* = 35) was introduced to obtain histological slides to evaluate immune cell infiltrative ranges.

**Results:**

Altogether six major lesion groups have been identified (A: 0% calcified, 0% liquefied; B: ≥50% calcified, 0% liquefied; C: < 50% calcified, < 50% liquefied; D: ≥50% calcified, < 50% liquefied; E: < 50% calcified, 50 ~ 75% liquefied; F: ≥50% calcified, ≥75% liquefied). Statistically, TBR values respectively were 5.1 ± 1.9, 2.7 ± 1.2, 4.2 ± 1.2, 2.7 ± 0.7, 4.6 ± 1.2, 2.9 ± 1.1 in groups A ~ F, and comparisons showed A > B, A > D, A > F, E > B, E > D, E > F, C > B, C > D, C > F (*P* < 0.05); LME ranges indicated by PET/CT respectively were 14.9 ± 3.9, 10.6 ± 1.5, 12.3 ± 1.1, 7.8 ± 1.6, 11.1 ± 2.3, 7.0 ± 0.4 mm in groups A ~ F, and comparisons showed A > B, A > D, A > F, A > E, C > B, C > D, C > F, E > D, E > F, B > D, B > F (*P* < 0.05); LME ranges indicated by MSS respectively were 17.9 ± 4.9, 13.0 ± 2.7, 11.9 ± 2.6, 6.0 ± 2.2, 11.0 ± 4.1, 6.0 ± 2.2 mm in groups A ~ F, and comparisons showed A > C, A > D, A > F, B > D, B > F, C > D, C > F (*P* < 0.05). Generally, less calcifications indicated higher TBR values and wider LME ranges; and, severer liquefactions indicated smaller LME ranges. Additionally, patients with previous medication history had lower TBR values.

**Conclusions:**

PET/CT and MSS method showed distinct TBRs and LME ranges for different calcifications and liquefactions. This study would be able to provide references for both surgical resections of lesions and more accurate sample acquisitions for basic research targeted to immunology.

**Supplementary Information:**

The online version contains supplementary material available at 10.1186/s12879-021-06366-3.

## Background

Human alveolar echinococcosis (AE), caused by the larva stage of *Echinococcus multilocularis* infection, is one of the lethal infectious diseases and causes severe organ damage [1, 2]. Radical resection with negative margin associated with anti-parasitic medication is considered as the best curable option [2–4]. Liver being the predominant target organ (> 90%), hepatic AE presents complexity considering the infiltrative growth pattern of the lesion, variant lesion morphology, different clinical stages, distinct biological activity of the parasitic lesion, and metabolic activity of the lesion microenvironment (LME).

Moreover, liver resection during surgery and its’ accuracy including LME is a potential prognosis-influencing aspect [3]. Besides, sample acquisition from LME liver and adjacent liver tissues (respectively for experimental and control groups) has been introduced to study immunology, fibrosis, pathophysiological changes, etc. of the host [5–8]. However, there was no clear evidence for such sampling method that could clearly differentiate the real experimental and control liver tissues by separating the immune cell infiltrative boundary. And, there is little known about the quantification of standard uptake value (SUV) of ^18^F-fluodeoxyglucose in position emission tomography/computed tomography (PET/CT) regarding different LME ranges in several heterogenic lesion types.

In this study, we collected surgical hepatic AE subjects’ preoperative computed tomography (CT), PET/CT, clinical data, and LME liver samples acquired through multi-site sampling method (MSS). Our primary research results concerning such sampling method for immunological study has been reported as “at least 2 cm distant from lesion” as a method to obtain adjacent normal liver tissue for controls previously [5]. Patients were divided into six groups based on lesion parameters focusing on calcification and liquefaction features. And, SUV data associated with histopathological slides were analyzed to assess the tumor to background ratio (TBR) of SUV and LME ranges to provide first-hand results of such setting.

## Methods

### Clinical patient enrollment

Using prospective and retrospective method, surgical AE patients were enrolled in this study. Inclusion criteria: (i) hepatic AE patients with definitive surgical indications; (ii) patients with both preoperative abdominal CT and ^18^F-FDG-PET/CT; (iii) patients with enough liver tissue for MSS for pathology during surgery. On the contrary, patients with chronic inflammatory diseases, autoimmune diseases, liver failure (grade Child C), immunocompromised situations, acute cholangitis or hepatitis, severe metabolic disorders were excluded.

During study period, 75 patients (32 males and 43 females) with 96 lesions were enrolled, average age was 36.7 ± 11.8 years, among them, 14 patients had previous medication history. Among subjects, 35 patients with relevant above two imaging examinations but without surgery were included for TBR analysis (Table 1).

**Table 1:** Patient demographics and lesion features

Group	Description		No	M/F	Age (year)	Size (cm)	Previous medication history	Lesion Location	Stage (I/II/IIIa/IIIb/IV)	MSS cases
	Calcification degree	Liquefaction degree								
A	(-)	(-)	12	6/6	36.8±8.0	9.7±4.7	0	RTS (3), RL (4), LLL (2), LTS (1), LL(2), ML(1)	0/3/2/2/5	7
B	(++)	(-)	14	8/6	40.0±15.3	7.7±2.3	5	RTS (3), RL (2), ML (4), LLL(3), RPL(1), LL(1)	1/1/5/5/2	5
C	(+)	(+)	16	7/9	34.4±12.5	14.0±5.2	2	RTS (6), ML (3), LL (1), LLL (2), LTS (2), RPL (3)	0/0/3/8/5	8
D	(++)	(+)	12	3/9	34.9±13.0	10.8±4.3	3	RTS (4), ML (2), RL(4), LTS(1), RPL(1)	2/1/0/7/2	5
E	(+)	(++)	12	4/8	39.0±10.1	10.4±4.7	1	RTS (3), RL (5), LL(2), RPL(2)	0/1/3/5/3	5
F	(++)	(++)	9	4/5	34.6±9.8	13.8±7.6	3	RTS (1), RL (3), LL (2), LTS (3)	0/0/0/4/5	5
Sum			75	32/43	36.7±11.8	11.0±5.2	14	RTS (20), RL (18), ML (10), LL (8), LLL (7), LTS (7), RPL (7)	3/6/13/31/22	35

*M/F* male/female ratio; Age and lesion size were presented with mean ± SD, *RL* right lobe, *RTS* right trisection, *LTS* left trisection, *LLL* left lateral lobe, *ML* medial lobe, *RPL* right posterior lobe, *LL* left lobe

### Abdominal CT

Abdominal CT examinations had been performed using previous methods form our institution [9]. All CT images were interpreted as part of the clinical examinations by two experienced radiologists that specialized in abdominal imaging, and a third senior radiological expert was invited for possible bifurcations.

**Fig. 1:**
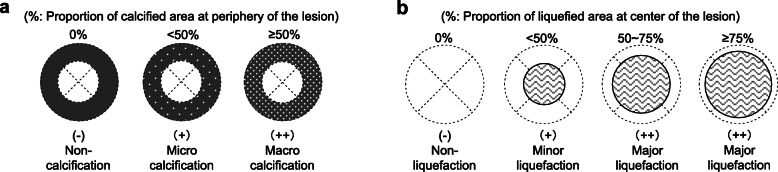
Semi-quantification of periphery calcification (white dots in gray background) and central liquefaction (wavy lines in black circle) of lesions (⊗ with dashed lines). **a** Calcification at lesion periphery was quantified mainly based on proportion of calcified area at periphery of the lesion and graded into three degrees [0%(−), < 50%(+) and ≥ 50%(++), respectively similar to non-calcification, micro calcification and macro calcifications]; **b** liquefaction at lesion center was quantified mainly based on proportion of liquefied area at center of the lesion and graded into four degrees [0%(−), < 50%(+), 50 ~ 75%(++) and ≥ 75%(++), respectively denoted as non-liquefaction, minor liquefaction and major liquefactions] according to liquefaction-to-lesion volumetric ratio

Basic parameters of AE lesions (size, location) and morphological features (calcification, liquefaction) were recorded in details. Diameter was measured in three dimensions (axis, coronary and sagittal) and average diameter was calculated as representative. Calcification at the lesion periphery was graded into three degrees [0%(−), < 50%(+) and ≥ 50%(++), respectively similar to non-calcification, micro-calcification and macro-calcifications [10]] (Fig. 1) no matter what the previously established calcification patterns [11] were, for the reason that those patterns were not specifically related to any clinical meaning except for accurate diagnosis of calcification [10]. Liquefaction-to-lesion volumetric ratios for each patient were calculated (Fig. S1), which then was used to grade liquefaction at lesion center into four degrees [0%(−), < 50%(+), 50 ~ 75%(++) and ≥ 75%(++)] according to liquefaction-to-lesion volumetric ratio that was measured using CT (Fig. 1).

### ^18^F-FDG-PET/CT, TBR and LME range indicated by PET/CT

Abdominal PET/CT examinations had been performed using previous methods form our institution [12]. Imaging diagnosis was carried out by two experienced physicians, and the PET, CT and PET/CT fusion images were independently analyzed: image quality was determined by visual analysis, allowing normal physiological uptake, as well as normal variations and artifacts; the value of maximum standardized uptake value (SUV_max_) of LME was measured after differentiating the biological borders of the lesions and the average SUV (SUV_ave_) of background liver at same slice was measured; TBR was calculated by SUV_max_ / SUV_ave_ (Fig. S2). Then, the value of ^18^F-FDG uptake area (*S*) and its’ length (*L*) were measured to calculate LME range indicated by PET/CT using *S/L* ratio (Fig. S3).

### Surgery and medical treatment

Patients were operated for hepatic AE after multidisciplinary team evaluation. Main procedures were conventional hepatectomy (minor, major, excessive), hepatectomy associated with vascular reconstruction, and ex vivo liver resection and autotransplantation for those contraindicated to conventional therapies [13], and one of them had received auxiliary partial autologous liver transplantation [14]. Post-operatively, all patients were enrolled in regular anti-parasitic medication therapy and followed up routinely according to expert consensus [1, 3].

### Pathology and LME range indicated by MSS method

MSS samples were acquired from surgical specimens. The specific sampling site were preoperatively planned through spatial information based on CT and PET/CT, even using three-dimensional reconstruction techniques [15, 16]. First, liver specimens which contained the target lesion (approximately 1 cm at thickness) as well as adherent liver tissue (at least for 5 cm at length) were cut into size about 2 cm*3 cm*6 cm, and fixed with formalin immediately. Note that, not all cases’ liver condition could allow sufficient liver tissue for MSS. For example, (a) left hemi-hepatectomy for left medial lobular lesions could allow MSS due to sufficient left lateral lobular liver parenchyma; (b) left lateral lobectomy for left lateral lobular “full-spaced” lesion could not allow for MSS because of insufficient adherent liver tissue at resection margin. Secondly, after 24–48 h fixation, MSS sampling was performed: liver samples were obtained at parallel levels to lesion surface with 5 mm intervals. Thirdly, paraffin embedded slides were prepared using routine protocols. At last, hematoxylin-eosin (HE) staining, immunohistochemistry (CD3, CD56 and CD68) and Masson staining were performed in order to determine the immune infiltrative areas’ range using manufacturers’ protocols. The widest range was defined by the most distant obviously positive expression of above three representative levels compared to even more distant slides (Fig. S4).

### Lesion categorization or grouping

Lesions were categorized into different groups constituting different degrees of calcifications and liquefactions according to relevant features. A demand for at least five repeats (at least five patients) for each group were set ahead of this study to assure the quality of statistical analysis.

### Statistical analysis

TBR and LME ranges were presented in median ± SD, and 95% CI were given. Non-parametric test (Mann Whitney test) were used to determine the significance of TBR and LME ranges between groups; linear regression was drawn to observe the link of TBR and LME ranges. *P* < 0.05 was chosen as standard to judge statistical significance.

## Results

### General baseline characteristics

Lesion size with average diameter was 11.0 ± 5.2 cm (ranging from 4.0–18.0 cm, larger lesion was chosen for multiple lesions in a same patient). Lesion location in the liver mainly was right trisection (20), right lobe (18), middle lobe (10), left lobe (8), left lateral lobe (7), left trisection (7) and right posterior lobe (7). According to expert consensus, clinical stages I, II, IIIa, IIIb and IV were respectively composed by 3, 6, 13, 31 and 22 cases (4.0, 8.0, 17.3, 41.3, 29.3%) [3]. CT and PET/CT were conducted by all subjects which had surgical treatment, but only 46.7% (*n* = 35) patients’ surgery allowed us to obtain MSS samples for pathology (Table 1).

### Patient grouping

**Fig. 2:**
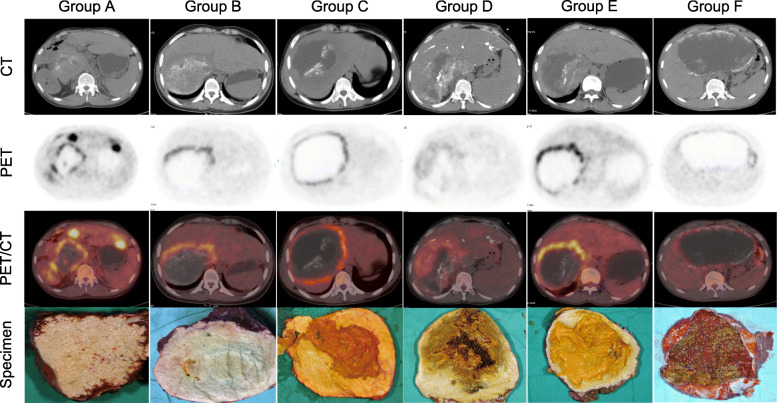
CT and PET/CT features plus gross specimens of patients in each groups. Group A: 0% periphery calcified (−/non-calcified), 0% central liquefied (−); Group B: ≥50% periphery calcified (++/macro-calcified), 0% central liquefied (−); Group C: < 50% periphery calcified (+/micro-calcified), < 50% central liquefied (+); Group D: ≥50% periphery calcified (++/macro-calcified), < 50% central liquefied (+); Group E: < 50% periphery calcified (+/micro-calcified), 50 ~ 75% central liquefied (++); Group F: ≥50% periphery calcified (++/macro-calcified), ≥75% central liquefied (++)

Altogether six major lesion groups have been identified (Fig. 2):

- Group A: 0% periphery calcified (−/non-calcified), 0% central liquefied (−);
- Group B: ≥50% periphery calcified (++/macro-calcified), 0% central liquefied (−);
- Group C: < 50% periphery calcified (+/micro-calcified), < 50% central liquefied (+);
- Group D: ≥50% periphery calcified (++/macro-calcified), < 50% central liquefied (+);
- Group E: < 50% periphery calcified (+/micro-calcified), 50 ~ 75% central liquefied (++);
- Group F: ≥50% periphery calcified (++/macro-calcified), ≥75% central liquefied (++).

**Fig. 3:**
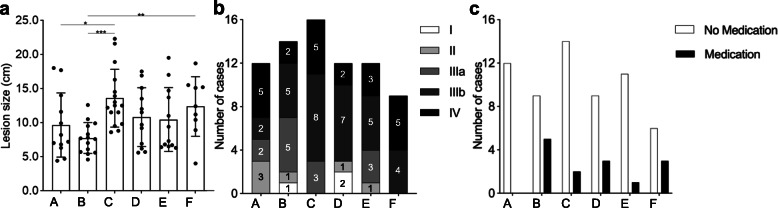
Lesion size, different clinical stages and previous medication history in each groups. **a** Lesion size in each groups (C > B, C > A, F > B, *P* < 0.05); **b** Different clinical stages in each groups (no apparent correlation or differences was observed between different clinical stages in different groups); **c** Previous medication history (albendazole) in each groups (except for Group A, no significance was observed between groups)

Of note, no other group of patients could be enrolled since there were no sufficient cases (less than three) for other constitutions of calcification and liquefaction. From the perspective of different groups, lesion size, clinical stage and previous medication history between each groups and their comparisons were presented in Fig. 3. Additionally, clinical stage was not apparently correlated to any degree of calcification or liquefaction. Each patients’ basic datasets for each groups have been presented in Supplementary Table 1.

### TBR values, PET/CT or MSS indicated LME ranges between each groups (Fig. 4a ~ c, Table 2)

TBR values in groups A ~ F respectively were 5.1 ± 1.9, 2.7 ± 1.2, 4.2 ± 1.2, 2.7 ± 0.7, 4.6 ± 1.2, 2.9 ± 1.1, and comparisons showed A > B, A > D, A > F, E > B, E > D, E > F, C > B, C > D, C > F (*P* < 0.05); Their 95% CI were 3.9 ~ 6.4, 2.0 ~ 3.4, 3.6 ~ 4.9, 2.3 ~ 3.2, 3.8 ~ 5.3, 2.1 ~ 3.8.

**Fig. 4:**
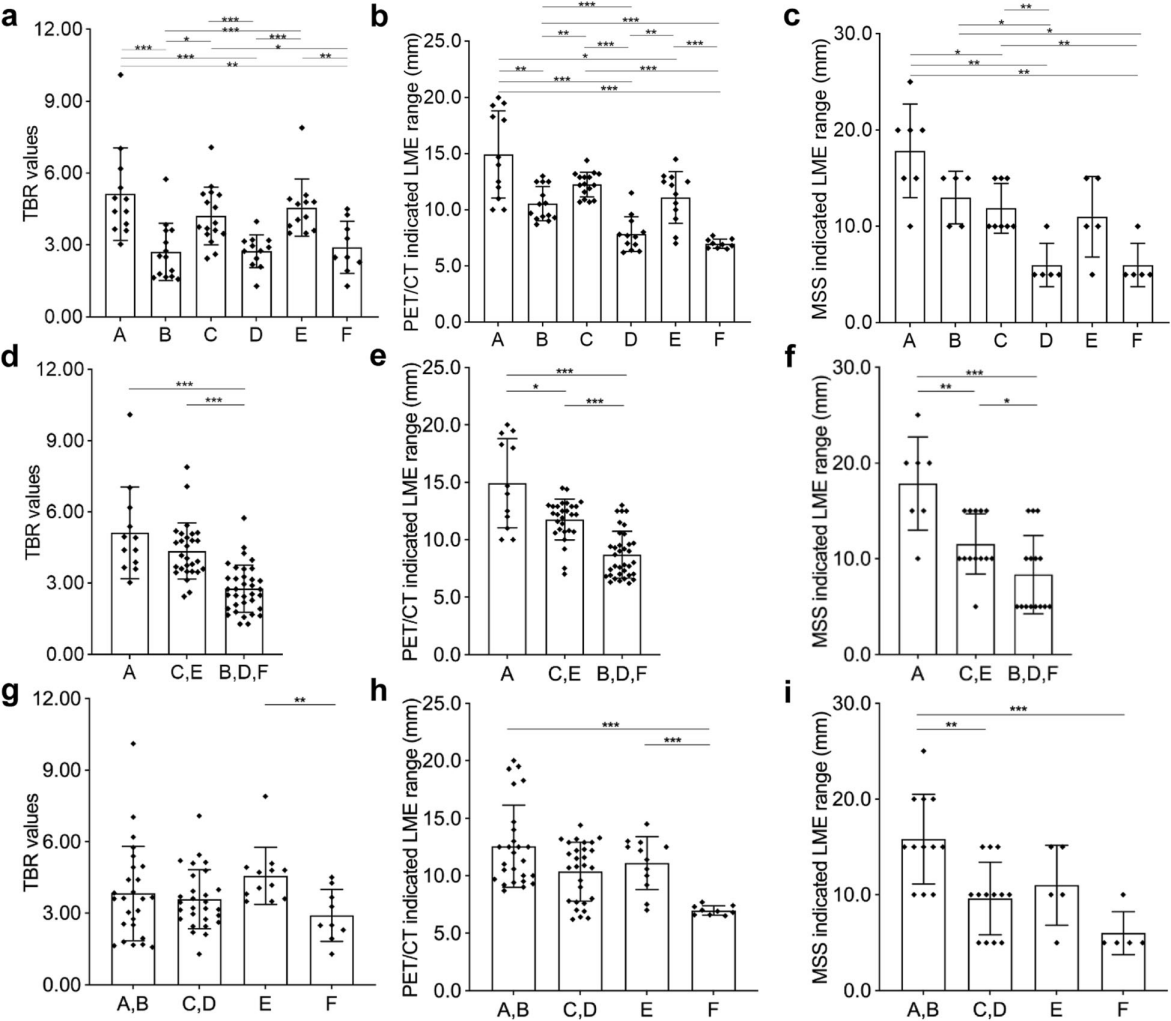
Comparisons of TBR values, PET/CT or MSS indicated LME ranges between different groups. **a** TBR values (A > B, A > D, A > F, E > B, E > D, E > F, C > B, C > D, C > F, *P* < 0.05); **b** PET/CT indicated LME range (A > B, A > D, A > F, A > E, C > B, C > D, C > F, E > D, E > F, B > D, B > F, *P* < 0.05); **c** MSS indicated LME range (A > C, A > D, A > F, B > D, B > F, C > D, C > F, *P* < 0.05); **d** TBR values from the perspective of different degrees of calcifications (A > B + D + F, C + E > B + D + F, *P* < 0.001); **e** PET/CT indicated LME range from the perspective of different degrees of calcifications (A > C + E, A > B + D + F, C + E > B + D + F, *P* < 0.05); **f** MSS indicated LME range from the perspective of different degrees of calcifications (A > C + E, A > B + D + F, C + E > B + D + F, *P* < 0.05); **g** TBR values from the perspective of different degrees of liquefactions (E > F, *P* < 0.01); **h** PET/CT indicated LME range from the perspective of different degrees of liquefactions (A + B > F, E > F, *P* < 0.001); **i** MSS indicated LME range from the perspective of different degrees of liquefactions (A + B > C + D, A + B > F, *P* < 0.01)

**Table 2:** Mean ± SD and 95% CI of TBR value and LME range indicated by PET/CT and MSS

Group	Description		TBR value		PET/CT indicated LME range (mm)		MSS indicated LME range (mm)	
	Calcification degree	Necrosis degree	Mean±SD	95% CI	Mean±SD	95% CI	Mean±SD	95% CI
A	(-)	(-)	5.1±1.9	3.9~6.4	14.9±3.9	12.5~17.4	17.9±4.9	13.3~22.4
B	(++)	(-)	2.7±1.2	2.0~3.4	10.6±1.5	9.7~11.4	13.0±2.7	9.6~16.4
C	(+)	(+)	4.2±1.2	3.6~4.9	12.3±1.1	11.7~12.9	11.9±2.6	9.7~14.0
D	(++)	(+)	2.7±0.7	2.3~3.2	7.8±1.6	6.8~8.8	6.0±2.2	3.2~8.8
E	(+)	(++)	4.6±1.2	3.8~5.3	11.1±2.3	9.6~12.6	11.0±4.1	5.8~16.2
F	(++)	(++)	2.9±1.1	2.1~3.8	7.0±0.4	6.7~7.3	6.0±2.2	3.2~8.8
C+E	(+)	Not specified	4.4±1.2	3.9~4.8	11.8±1.8	11.1~12.5	11.5±3.2	9.6~13.4
B+D+F	(++)	Not specified	2.8±1.0	2.4~3.1	8.7±2.0	8.0~9.4	8.3±4.1	6.1~10.6
A+B	Not specified	(-)	3.8±1.9	3.0~4.6	12.6±3.6	11.1~14.0	15.8±4.7	12.9~18.8
C+D	Not specified	(+)	3.6±1.2	3.1~4.1	10.4±2.6	9.4~11.4	9.6±3.8	7.3~11.9
Sum (A~F)			3.7±1.6	3.4~4.1	10.8±3.2	10.1~11.6	11.4±5.2	9.6~13.2

*TBR* tumor-to-background ratio of maximum standard uptake value of LME and background liver, *LME* lesion microenvironment, *MSS* multi-stie sampling method, *SD* standard division, *95% CI* 95% confidence interval of the mean

PET/CT indicated LME range in groups A ~ F respectively were 14.9 ± 3.9, 10.6 ± 1.5, 12.3 ± 1.1, 7.8 ± 1.6, 11.1 ± 2.3, 7.0 ± 0.4 mm in groups A ~ F, and comparisons showed A > B, A > D, A > F, A > E, C > B, C > D, C > F, E > D, E > F, B > D, B > F (*P* < 0.05); Their 95% CI were 12.5 ~ 17.4, 9.7 ~ 11.4, 11.7 ~ 12.9, 6.8 ~ 8.8, 9.6 ~ 12.6, 6.7 ~ 7.3 mm.

MSS indicated LME range in groups A ~ F respectively were 17.9 ± 4.9, 13.0 ± 2.7, 11.9 ± 2.6, 6.0 ± 2.2, 11.0 ± 4.1, 6.0 ± 2.2 mm in groups A ~ F, and comparisons showed A > C, A > D, A > F, B > D, B > F, C > D, C > F (*P* < 0.05); Their 95% CI were 13.3 ~ 22.4, 9.6 ~ 16.4, 9.7 ~ 14.0, 3.2 ~ 8.8, 5.8 ~ 16.2, 3.2 ~ 8.8 mm.

### TBR values, PET/CT or MSS indicated LME ranges from the perspective of different degrees of calcifications (Fig. 4d ~ f, Table 2)

TBR values in groups A, C + E and B + D + F respectively were 5.1 ± 1.9, 4.4 ± 1.2, 2.8 ± 1.0, and comparisons showed A > B + D + F, C + E > B + D + F (*P* < 0.001); Their 95% CI were 3.9 ~ 6.4, 3.9 ~ 4.8, 2.4 ~ 3.1.

PET/CT indicated LME ranges in groups A, C + E and B + D + F respectively were, 14.9 ± 3.9, 11.8 ± 1.8, 8.7 ± 2.0, and comparisons showed A > C + E, A > B + D + F, C + E > B + D + F, (*P* < 0.05); Their 95% CI were 12.5 ~ 17.4, 11.1 ~ 12.5, 8.0 ~ 9.4.

MSS indicated LME ranges in groups A, C + E and B + D + F respectively were 17.9 ± 4.9, 11.5 ± 3.2, 8.3 ± 4.1, and comparisons showed A > C + E, A > B + D + F, C + E > B + D + F, (*P* < 0.05); Their 95% CI were 13.3 ~ 22.4, 9.6 ~ 13.4, 6.1 ~ 10.6.

### TBR values, PET/CT or MSS indicated LME ranges from the perspective of different degrees of liquefactions (Fig. 4g ~ i, Table 2)

TBR values in groups A + B, C + D, E and F respectively were 3.8 ± 1.9, 3.6 ± 1.2, 4.6 ± 1.2, 2.9 ± 1.1, and comparisons showed E > F (*P* < 0.01); Their 95% CI were 3.0 ~ 4.6, 3.1 ~ 4.1, 3.8 ~ 5.3, 2.1 ~ 3.8.

PET/CT indicated LME ranges in groups A + B, C + D, E and F respectively were 12.6 ± 3.6, 10.4 ± 2.6, 11.1 ± 2.3, 7.0 ± 0.4, and comparisons showed A + B > F, E > F (*P* < 0.001); Their 95% CI were 11.1 ~ 14.0, 9.4 ~ 11.4, 9.6 ~ 12.6, 6.7 ~ 7.3 mm.

MSS indicated LME ranges in groups A + B, C + D, E and F respectively were 15.8 ± 4.7, 9.6 ± 3.8, 11.0 ± 4.1, 6.0 ± 2.2, and comparisons showed A + B > C + D, A + B > F (*P* < 0.01); Their 95% CI were 12.9 ~ 18.8, 7.3 ~ 11.9, 5.8 ~ 16.2, 3.2 ~ 8.8 mm

### Correlations between TBR values, PET/CT or MSS indicated LME ranges

**Fig. 5:**
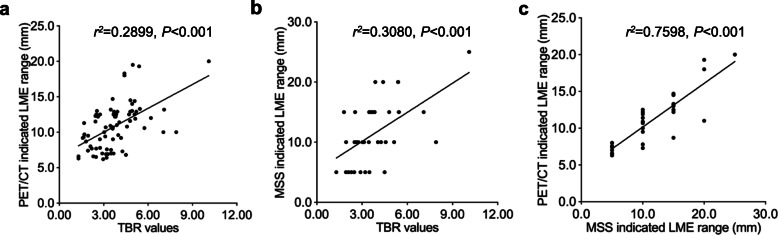
Correlations between TBR values and LME ranges. **a** TBR values was positively correlated to PET/CT indicated LME range (*r*^*2*^ = 0.2899, *P* < 0.001); **b** TBR values was positively correlated to MSS indicated LME range (*r*^*2*^ = 0.3080, *P* < 0.001); **c** MSS indicated LME range was positively correlated to PET/CT indicated LME range (*r*^*2*^ = 0.7598, *P* < 0.001)

In order to further understand the correlations between relevant results, linear correlations were drawn: LME ranges indicated by PET/CT and MSS were positively correlated to TBR values, and their relevant *r*^*2*^ and *P* values were (*r*^*2*^ = 0.2899, *P* < 0.001) and (*r*^*2*^ = 0.3080, *P* < 0.001); besides, LME ranges indicated by PET/CT was also positively correlated to TBR values (*r*^*2*^ = 0.7598, *P* < 0.001) (Fig. 5).

### TBR values, PET/CT or MSS indicated LME ranges and previous medication history of patients

**Fig. 6:**
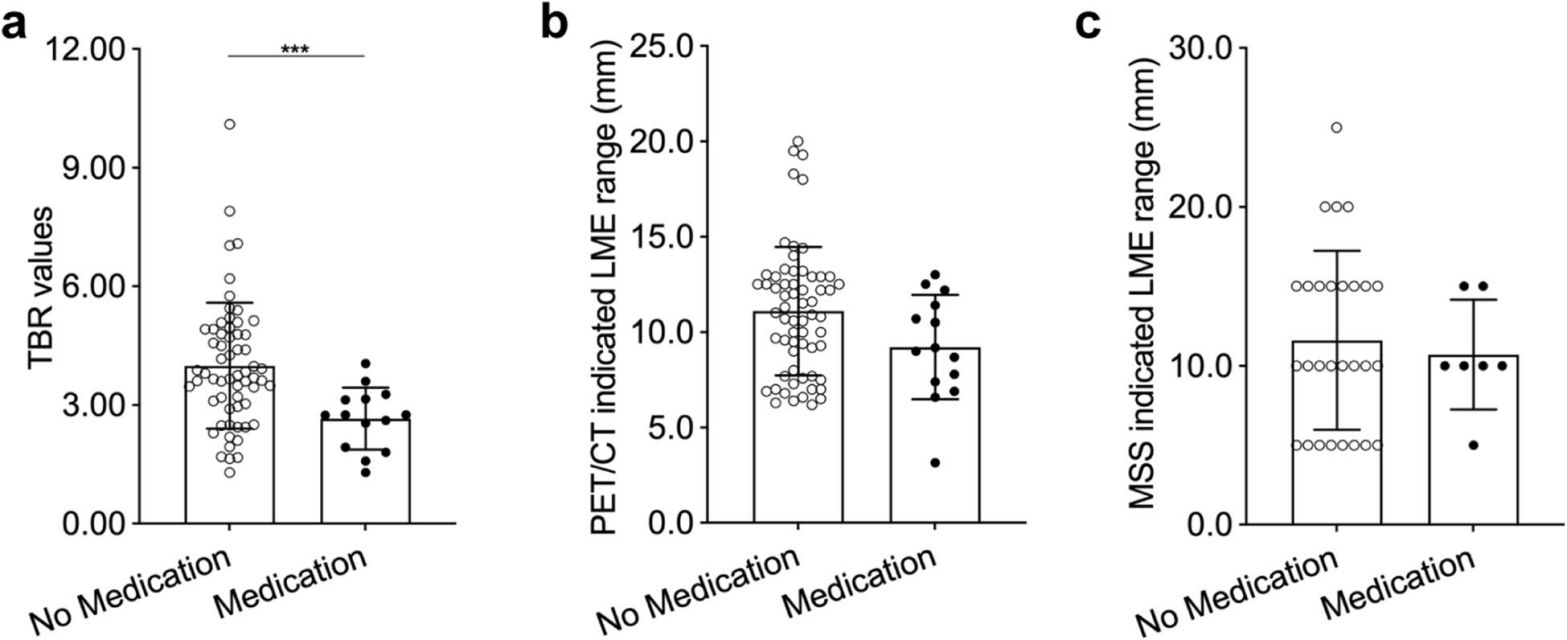
Comparisons of TBR values, PET/CT or MSS indicated LME ranges between without and with previous medication history. **a** TBR values was smaller in patients with previous medication history than without previous medication history (*P* < 0.001); **b** PET/CT indicated LME range (no statistical significance); **c** MSS indicated LME range (no statistical significance)

In order to understand the influence of previous medication history on above values, clustered analysis was performed. However, the only significant result was that TBR values was smaller in patients who had previous medication (albendazole) history (Fig. 6). In addition, lesion size and different clinical stages were also analyzed to see if there were any correlations between them and above three values. But, no valuable results were observed, indicating that lesion size and different clinical stages had impact on none of the above three aspects of a lesion (Fig. S5, Fig. S6).

## Discussion

Chronic and infiltrative growth pattern of AE lesion could result into many pathophysiological changes within the liver [4]. We have noticed the spatial heterogeneity of AE lesions at early times, and practiced this idea much from the perspectives of diagnosis, clinical treatment and basic researches. Obviously, detailed lesion features could help radiologists, clinicians, parasitologists to precisely evaluate and choose certain management options for patients [17–19]. AE lesion includes various factors to be evaluated during the whole process: basic or anatomical characteristics (size or volume, location, ect.), morphology (by radiology and pathological view), calcification features (by radiology mostly), lesion biological activity (or parasitic viability), LME metabolic activity (mostly assessed by PET/CT so far), vasculature involvements (both intra- and extra-hepatic vessels including biliary trees, these are also linked to certain comorbidities that to be assessed), immunological status of the patients. Among them, LME has core roles from the perspectives of anatomy, radiology, pathophysiology, and immunology.

In the past, several imaging tools demonstrated lesion types for hepatic AE [11, 20, 21]. And there were some other imaging methods and researches about comparison of different imaging tools [10, 22–24]. These strategies had proposed new methods for lesion categorization. Nevertheless, no integrative typing system has been established so far, and they were asymmetric when defining clinical stages or lesion activity regarding different lesion types. Besides, among the lesion types that were proposed by *Kodama* et al, *Kratzer* et al and *Graeter* et al, there were not clear integrative bridge to definitely link them, and no relevant study that used all these imaging tools in same patient cohort has been reported [11, 20, 21]. Basically, *Graeter* et al defined different calcification patterns very precisely so far. However, no specific correlations between such classification of calcifications and lesion activity, or definitive clinical meaning were drawn in their study or other researches, as French professionals pointed out [10]. Moreover, it is the lesion periphery where the parasite-host interactions happen far more than lesion center, from our point of view. Therefore, we semi-quantified calcification at lesion periphery in our study, somehow, which was a notable progress.

At present, PET/CT is the most valuable tool to assess perilesion liver metabolic activity (or parasitic lesion activity indirectly) and seems to be an unreplaceable imaging method [1, 10]. At the meantime, calcification and liquefaction could be evaluated by CT easily. Besides, we developed MSS method to assess representative immune cell infiltration ranges for the first time, and we believe that it will be a useful tool to accurately evaluate immune infiltration range in the future. It is understandable that calcification and liquefaction are two different ways of parasitic death, and they offer valuable information about clinical stages. Different degrees of calcification and liquefaction as well as integration of them can reveal further insights of AE lesions.

In current study, we assessed TBR regarding different lesions that featured with semi-quantified calcification and liquefaction, which were further evidenced with gross specimens and microscopic analysis. Our results showed that there were significant TBR differences between different groups, as well as different degrees of calcification and liquefactions (Fig. 4). Apparently, less calcified lesions had higher TBR values. The higher TBR value was, the stronger the lesion activity. Interestingly, LME ranges also varied between different groups of patients. Results also evidenced that higher TBR ranges presented wider LME ranges within certain limits (Fig. 5a, b). It was worth to point out that, even the TBRs was very high, LME range could not be symmetrically wide, because parasitic-host interactions need close contact, and the longer the distance was, the harder the enrichment of host cells or other parenchymal components happen. When it came to LME range (^18^F-FDG uptake region around the lesion), PET/CT and MSS presented similar results (Fig. 5c, Table 2). Besides, previous medication history was statistically significant only in TBR value comparisons rather than LME ranges (Fig. 6).

This study could provide references in sampling in basic researches and surgical resection:

1. Practically, the LME region may directly affect the experimental outcome when it's not balanced properly by mimicking real experimental liver tissue with the real control liver tissues. A previous research has studied surrogate markers in distinguishing metabolically active and inactive AE patients [25]. In this study, methodology for sampling was defined as “specimens were taken from the AE lesion area and from a macroscopically normal distant area of liver tissue”, although the precise area had not been described priorily, there was a well consideration for control liver tissue acquisition based on pathology. However, another recent study sampled the liver tissues using 2 cm line to study Kupffer cell and fibrosis in hepatic AE [7]. But, no specific leison heterogeniety was decribed except for an expression that “the liver tissues were taken within 2 cm of the lesion by surgery for the close group, whereas the liver tissues were taken 2 cm outside the lesion for the distance group”. If a 2 cm line has to be chosen to determine the experimental and control liver tissues: (a) it would definitely reduce both of the targeted immune cell number and cell types in cases with narrower LME range than 2 cm; (b) it would increase them in cases with wider LME range than 2 cm. Meanwhile, some other studies never layed out the sampling areas or scales [26]. Of course, it was major result-influencing factor. So, we strongly recommend that the differentiative line should be based on different lesion types to achieve best performance of liver-based basic studies for AE (Table 2).
2. The potential for resection and whether there is disease dissemination must be assessed carefully by pre-operative imaging techniques [3]. Surgical removal of parasitic lesion plus peri-lesion inflammatory belt was recommended. A western study demonstrated that R0 resection had 2% disease recurrence, whereas, R1 and R2 resections showed 41% of intrahepatic disease progression during postsurgical follow-up [27]. Another study reported late disease recurrence even after R0 resection [28]. Thus,  the importance of R0 resection of AE lesion was emphasized. However, how wide the peri-lesion inflammatory belt (corresponding to LME) has not been studied based on distinct lesion types. Besides, achieving 2 cm resection margin for every single lesion is not possible in most advanced cases as recommended [3, 4, 27, 29]. For excessive vascular-infiltrated lesion or with severe comorbidities, only liver transplantation or ex vivo liver resection associated with autotransplantation could be selected from the perspective of surgical treatment [13, 30–33]. Our data indicated that, different lesion types had different immune cell infiltrated belt. For major/obvious calcified lesions, less than 2 cm resection margin would be satisfactory; for severe liquefied lesion with obvious calcified capsule, less than 1 cm resection margin would be enough. Extra indications for resection margin could also be concluded from our study results based on 95% CI (Table 2).

Objectively speaking, shortcomings of this study were that we were short when enrolling patients which had both PET/CT and MSS data because of different surgical approaches (not all surgeries could provide sufficient liver samples), and some other lesion categories were not included due to unavailability of PET/CT or MSS. Moreover, MSS with 5 mm intervals off the lesion shore prevented us to map the immune cell infiltration with higher resolution. Further, methodology for simulating every spatial SUV digital data would be more helpful to understand the spatial heterogeneity of a lesion. Further insights of LME should be discovered in depth in future researches. Further studies may add much value if the root−/coral-like tubovesicular or vesicotubular structures [34] of the parasite into LME liver is identified based on different lesion categories, and which will be helpful when deciding pathologically radical resection margins, at that time, resection of immune ifiltrated belt might not be needed.

## Conclusions

This pioneering study would be able to provide references for both surgical removal of lesions and sample acquisitions more accurately for basic research targeted to immunology and pathophysiological changes of LME. Less calcifications indicated higher TBR values and wider LME ranges; PET/CT and MSS had similar discoverability for LME ranges, which varies among different lesion groups. Sample acquisition based on different lesion types were strongly advised for certain experimental and control studies.

## Supplementary Information

**Additional file 1: Supplementary Table 1.** Data resources.

**Additional file 2: Figure S1.** Liquefaction-to-lesion volumetric ratio (in the order of small to large values) of all patients.

**Additional file 3: Figure S2.** Calculation of TBR value based on SUV measurements.

**Additional file 4: Figure S3.** Calculation of LME range indicated by PET.

**Additional file 5: Figure S4.** Representative immune cell infiltration in each groups and quantification of LME range using MSS method.

**Additional file 6: Figure S5.** Distribution of TBR values, PET/CT and MSS indicated LME ranges regarding lesion size.

**Additional file 7: Figure S6.** Distribution of TBR values, PET/CT and MSS indicated LME ranges regarding different clinical stages.

## Data Availability

The datasets used/analyzed during the current study were within the manuscript, and further data could be available from the corresponding author on reasonable requests.

## Abbreviations

AE: Alveolar echinococcosis
LME: Lesion microenvironment
PET/CT: Position emission tomography and computed tomography
MSS: Multi-site sampling method
TBRs: Tumor to background ratios
SUV: Standard uptake value
^18^F-FDG: ^18^F-fluodeoxyglucose
RL: Right lobe of the liver
RTS: Right trisection of the liver
LTS: Left trisection of the liver
LLL: Left lateral lobe of the liver
ML: Medial lobe of the liver
RPL: Right posterior lobe of the liver
R0 Resection: Radial resection with pathologically negative margin
PNM: P for parasitic lesion in the liver, N for neighboring organ, M for metastasis, they together indicate clinical stages of AE according to the expert consensus
