# Supplementary material for: Quantitative evaluation of range and metabolic activity of hepatic alveolar echinococcosis lesion microenvironment using PET/CT and multi-site sampling method
Source: BMC Infect Dis. 2021 Jul 23;21:702. doi: 10.1186/s12879-021-06366-3 (PMC8299608; doi:10.1186/s12879-021-06366-3)
Supplement: Supplementary file 1 — Additional file 1: Supplementary Table 1. Data resources. [file 12879_2021_6366_MOESM1_ESM.docx]

**Supplementary Table 1.** Data resources.

| No | Group | Lesion size (cm) | Lesion location | PNM classification | Clinical stage | Medication history | Data availability | | TBR value | LME range (mm) indicated by: | |
| --- | --- | --- | --- | --- | --- | --- | --- | --- | --- | --- | --- |
|  |  |  |  |  |  |  | PET/CT | MSS |  | PET/CT | MSS |
| 1 | A | 4.7, 3.5 | RL | P_3_M_1_N_1_ | IV | No | Yes | Yes | 4.40 | 18.0 | 20.0 |
| 2 | A | 11.6, 3.6, 2.5 | RL, LLL | P_4_M_1_N_0_ | IV | No | Yes | Yes | 10.11 | 20.0 | 25.0 |
| 3 | A | 18.0 | LTS | P_4_M_1_N_1_ | IV | No | Yes | Yes | 3.66 | 12.5 | 15.0 |
| 4 | A | 9.9 | RL | P_2_M_0_N_0_ | II | No | Yes | No | 4.96 | 19.5 | - |
| 5 | A | 14.0 | RTS | P_2_M_0_N_0_ | II | No | Yes | Yes | 3.88 | 11.0 | 20.0 |
| 6 | A | 11.6 | RTS | P_4_M_0_N_0_ | IIIb | No | Yes | No | 7.03 | 10.0 | - |
| 7 | A | 7.2 | RTS | P_4_M_1_N_0_ | IV | No | Yes | Yes | 3.03 | 10.0 | 10.0 |
| 8 | A | 4.7 | ML | P_2_M_0_N_0_ | II | No | Yes | Yes | 5.39 | 19.3 | 20.0 |
| 9 | A | 5.1, 1.7 | LLL | P_3_M_1_N_1_ | IV | No | Yes | No | 4.40 | 18.3 | - |
| 10 | A | 5.8, 1.2 | LL | P_3_M_0_N_0_ | IIIa | No | Yes | Yes | 3.60 | 14.7 | 15.0 |
| 11 | A | 6.3 | RL | P_3_M_0_N_0_ | IIIa | No | Yes | No | 4.92 | 12.0 | - |
| 12 | A | 4.4 | LL | P_2_M_1_N_0_ | IIIb | No | Yes | No | 6.19 | 14.0 | - |
| 13 | B | 7.7 | ML | P_1_M_0_N_0_ | I | Yes | Yes | No | 2.75 | 12.5 | - |
| 14 | B | 6.1 | ML | P_2_M_1_N_0_ | IIIb | No | Yes | No | 1.69 | 9.7 | - |
| 15 | B | 7.0 | ML | P_4_M_1_N_0_ | IV | Yes | Yes | Yes | 2.55 | 13.0 | 15.0 |
| 16 | B | 7.6 | RL | P_3_M_0_N_0_ | IIIa | No | Yes | No | 1.67 | 11.3 | - |
| 17 | B | 7.2, 3.3 | RTS | P_3_M_0_N_0_ | IIIa | Yes | Yes | No | 3.60 | 10.5 | - |
| 18 | B | 8.3 | RTS | P_3_M_0_N_0_ | IIIa | No | Yes | Yes | 2.50 | 11.2 | 10.0 |
| 19 | B | 12.6 | RTS | P_4_M_1_N_1_ | IV | No | Yes | Yes | 3.84 | 11.2 | 15.0 |
| 20 | B | 5.6 | RPL | P_2_M_0_N_0_ | II | No | Yes | No | 5.75 | 10.6 | - |
| 21 | B | 7.8 | LLL | P_3_M_0_N_0_ | IIIa | No | Yes | Yes | 1.94 | 9.5 | 10.0 |
| 22 | B | 5.7 | LL | P_3_M_0_N_0_ | IIIa | Yes | Yes | No | 1.58 | 9.2 | - |
| 23 | B | 4.6 | LLL | P_2_M_1_N_0_ | IIIb | Yes | Yes | Yes | 1.80 | 8.7 | 15.0 |
| 24 | B | 9.9 | ML | P_2_M_1_N_0_ | IIIb | No | Yes | No | 3.62 | 9.0 | - |
| 25 | B | 5.4 | LLL | P_2_M_1_N_0_ | IIIb | No | Yes | No | 3.10 | 9.4 | - |
| 26 | B | 9.5 | RL | P_3_M_1_N_0_ | IIIb | No | Yes | No | 1.64 | 9.3 | - |
| 27 | C | 13.0 | LL | P_3_M_0_N_0_ | IIIa | No | Yes | Yes | 4.57 | 10.8 | 10.0 |
| 28 | C | 15.7, 12.6 | LL | P_4_M_1_N_0_ | IV | No | Yes | Yes | 3.47 | 13.2 | 15.0 |
| 29 | C | 9.8 | ML | P_3_M_0_N_0_ | IIIa | No | Yes | Yes | 5.44 | 13.3 | 15.0 |
| 30 | C | 13.9 | RTS | P_3_M_0_N_0_ | IIIa | No | Yes | Yes | 7.08 | 13.2 | 15.0 |
| 31 | C | 14.5 | RTS | P_3_M_0_N_1_ | IV | No | Yes | No | 3.76 | 12.2 | - |
| 32 | C | 13.9, 5.0 | RTS, LLL | P_3_M_1_N_0_ | IIIb | Yes | Yes | Yes | 3.13 | 10.7 | 10.0 |
| 33 | C | 11.5 | LTS | P_4_M_0_N_0_ | IIIb | No | Yes | No | 3.69 | 12.9 | - |
| 34 | C | 11.5 | ML | P_4_M_0_N_0_ | IIIb | No | Yes | Yes | 5.13 | 11.9 | 10.0 |
| 35 | C | 9.5 | ML | P_4_M_0_N_0_ | IIIb | Yes | Yes | Yes | 2.61 | 12.2 | 10.0 |
| 36 | C | 12.8, 8.8 | RPL, LLL | P_4_M_1_N_0_ | IV | No | Yes | Yes | 3.45 | 10.9 | 10.0 |
| 37 | C | 13.2, 3.1 | RTS | P_4_M_1_N_0_ | IV | No | Yes | No | 4.78 | 11.6 | - |
| 38 | C | 12.5 | RL | P_4_M_1_N_0_ | IV | No | Yes | No | 2.44 | 12.3 | - |
| 39 | C | 8.8 | RL, LLL | P_3_M_1_N_0_ | IIIb | No | Yes | No | 5.09 | 14.4 | - |
| 40 | C | 8.6 | LTS | P_3_M_1_N_0_ | IIIb | No | Yes | No | 3.92 | 12.9 | - |
| 41 | C | 12.5 | RTS | P_4_M_0_N_0_ | IIIb | No | Yes | No | 3.61 | 10.7 | - |
| 42 | C | 12.2 | RTS | P_4_M_0_N_0_ | IIIb | No | Yes | No | 5.20 | 12.9 | - |
| 43 | D | 9.2 | ML | P_4_M_0_N_0_ | IIIb | Yes | Yes | No | 2.74 | 9.0 | - |
| 44 | D | 14.0, 3.5 | RTS | P_3_M_1_N_0_ | IIIb | No | Yes | Yes | 3.21 | 7.6 | 5.0 |
| 45 | D | 14.9, 2.0 | RTS | P_4_M_0_N_0_ | IIIb | Yes | Yes | Yes | 2.75 | 7.8 | 10.0 |
| 46 | D | 8.0, 4.1, 3.0 | RTS | P_4_M_1_N_1_ | IV | No | Yes | Yes | 2.90 | 7.0 | 5.0 |
| 47 | D | 12.0 | RTS | P_4_M_0_N_0_ | IIIb | No | Yes | No | 3.98 | 9.6 | - |
| 48 | D | 5.7 | ML | P_1_M_0_N_0_ | I | No | Yes | Yes | 2.11 | 8.0 | 5.0 |
| 49 | D | 5.6 | LTS | P_1_M_0_N_0_ | I | No | Yes | No | 2.44 | 11.5 | - |
| 50 | D | 6.2 | RL | P_2_M_0_N_0_ | II | Yes | Yes | No | 3.15 | 6.9 | - |
| 51 | D | 13.5 | RL | P_4_M_1_N_0_ | IV | No | Yes | No | 2.96 | 6.2 | - |
| 52 | D | 6.9 | RPL | P_3_M_1_N_0_ | IIIb | No | Yes | No | 3.19 | 6.4 | - |
| 53 | D | 9.7 | RL | P_4_M_0_N_0_ | IIIb | No | Yes | Yes | 1.29 | 6.3 | 5.0 |
| 54 | D | 11.5 | RL | P_4_M_0_N_0_ | IIIb | No | Yes | No | 2.19 | 7.7 | - |
| 55 | E | 13.7 | RL | P_4_M_1_N_0_ | IV | No | Yes | Yes | 4.81 | 14.5 | 15.0 |
| 56 | E | 11.0 | RL | P_4_M_1_N_0_ | IV | No | Yes | Yes | 7.90 | 10.0 | 10.0 |
| 57 | E | 17.0 | RTS | P_3_M_0_N_0_ | IIIa | No | Yes | No | 3.49 | 7.0 | - |
| 58 | E | 14.7 | RTS | P_2_M_1_N_1_ | IV | No | Yes | Yes | 3.49 | 7.5 | 5.0 |
| 59 | E | 16.7, 2.8 | RTS | P_4_M_0_N_0_ | IIIb | No | Yes | Yes | 3.80 | 12.2 | 15.0 |
| 60 | E | 6.3 | RPL | P_2_M_0_N_0_ | II | No | Yes | No | 5.08 | 12.5 | - |
| 61 | E | 6.7 | LL | P_3_M_0_N_0_ | IIIa | Yes | Yes | Yes | 4.06 | 11.4 | 10.0 |
| 62 | E | 6.5 | LL | P_3_M_0_N_0_ | IIIa | No | Yes | No | 4.71 | 12.5 | - |
| 63 | E | 5.3, 1.5 | RL | P_2_M_1_N_0_ | IIIb | No | Yes | No | 4.79 | 12.9 | - |
| 64 | E | 6.4 | RL | P_2_M_1_N_0_ | IIIb | No | Yes | No | 4.92 | 13.0 | - |
| 65 | E | 8.3, 1.1 | RPL | P_3_M_1_N_0_ | IIIb | No | Yes | No | 4.17 | 9.2 | - |
| 66 | E | 7.3 | RL | P_4_M_0_N_0_ | IIIb | No | Yes | No | 3.60 | 10.6 | - |
| 67 | F | 4.0 | LL | P_2_M_1_N_0_ | IIIb | Yes | Yes | Yes | 1.93 | 7.4 | 5.0 |
| 68 | F | 6.8, 3.6 | LL | P_3_M_1_N_1_ | IV | No | Yes | No | 3.66 | 7.0 | - |
| 69 | F | 15.5 | LTS | P_4_M_0_N_0_ | IIIb | No | Yes | Yes | 2.48 | 6.5 | 5.0 |
| 70 | F | 12.5, 2.1, 4.1 | RL | P_2_M_0_N_1_ | IV | No | Yes | No | 2.49 | 7.7 | - |
| 71 | F | 15.2 | RTS | P_4_M_1_N_1_ | IV | No | Yes | Yes | 4.27 | 7.3 | 10.0 |
| 72 | F | 11.2 | RL | P_4_M_1_N_0_ | IV | No | Yes | Yes | 2.29 | 6.6 | 5.0 |
| 73 | F | 12.5 | LTS | P_4_M_1_N_0_ | IV | Yes | Yes | - | 1.29 | 6.6 | - |
| 74 | F | 15.1 | LTS | P_3_M_1_N_0_ | IIIb | No | Yes | Yes | 4.50 | 6.8 | 5.0 |
| 75 | F | 8.9 | RL | P_3_M_1_N_0_ | IIIb | Yes | Yes | - | 3.27 | 6.9 | - |

Note: (1) Abbreviations: PET/CT= positron emission tomography and computed tomography; MSS=multi-site sampling method; TBR=tumor-to-background ratio (no unit); LME=lesion microenvironment; RL=right lobe hemi-liver; RPL=right posterior lobe liver; RTS=right trisection (right lobe plus left medial lobe liver); ML=middle lobe liver (left medial lobe liver); LLL=left lateral lobe liver; LL=left lobe hemi-liver (ML+LLL); LTS=left trisection (LL plus right anterior lobe liver); A-F=groups representatives.

(2) Explanations: Lesion size was recorded with average diameter of each single lesion; PNM classifications and clinical stages were based on WHO/IWGE recommendations.
