# Supplementary material for: Quantitative evaluation of range and metabolic activity of hepatic alveolar echinococcosis lesion microenvironment using PET/CT and multi-site sampling method
Source: BMC Infect Dis. 2021 Jul 23;21:702. doi: 10.1186/s12879-021-06366-3 (PMC8299608; doi:10.1186/s12879-021-06366-3)
Supplement: Supplementary file 2 — Additional file 2: Figure S1. Liquefaction-to-lesion volumetric ratio (in the order of small to large values) of all patients. [file 12879_2021_6366_MOESM2_ESM.pptx]

## Slide 1
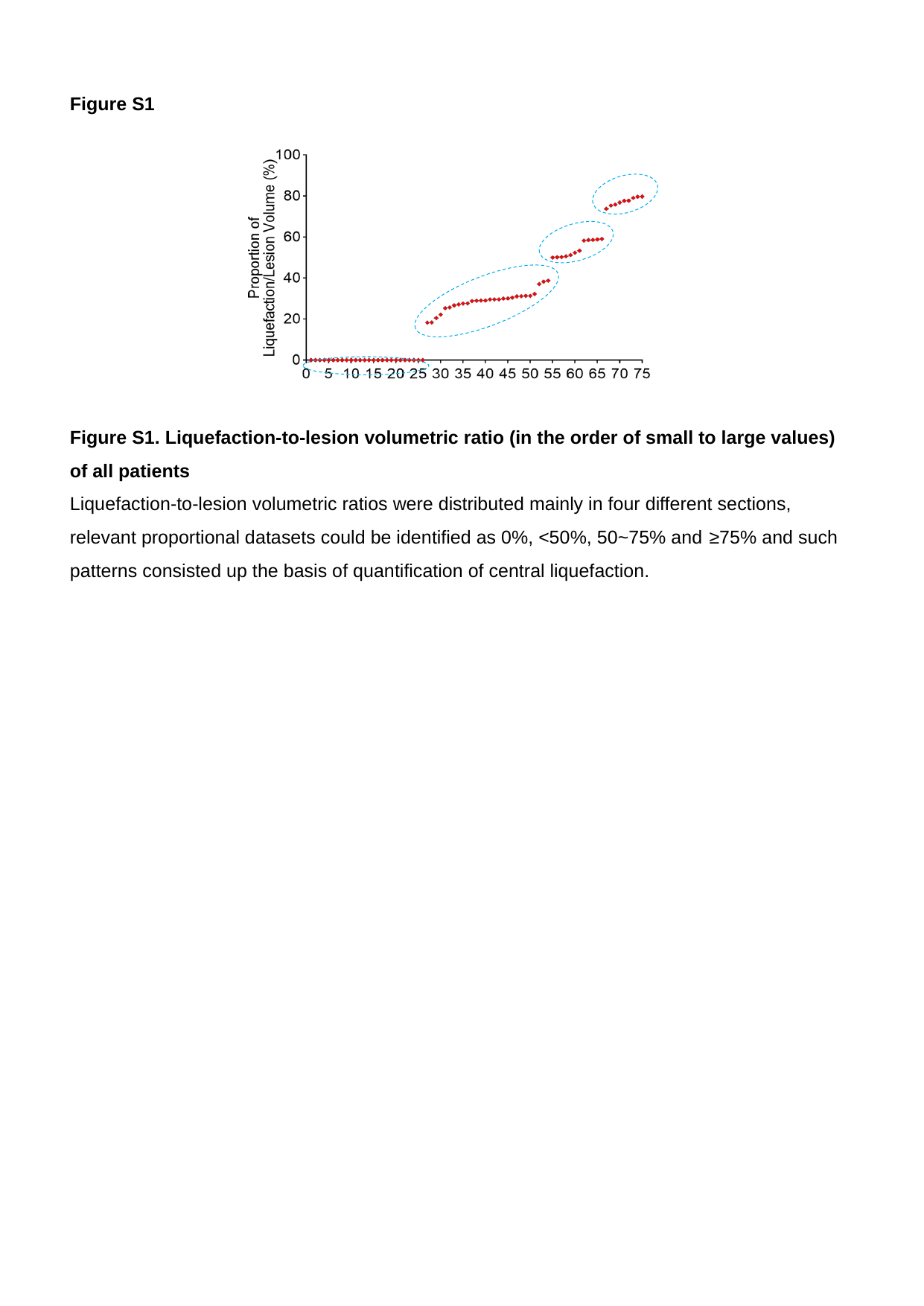

Figure S1
Figure S1. Liquefaction-to-lesion volumetric ratio (in the order of small to large values) of all patients
Liquefaction-to-lesion volumetric ratios were distributed mainly in four different sections, relevant proportional datasets could be identified as 0%, <50%, 50~75% and ≥75% and such patterns consisted up the basis of quantification of central liquefaction.
