# Supplementary material for: Quantitative evaluation of range and metabolic activity of hepatic alveolar echinococcosis lesion microenvironment using PET/CT and multi-site sampling method
Source: BMC Infect Dis. 2021 Jul 23;21:702. doi: 10.1186/s12879-021-06366-3 (PMC8299608; doi:10.1186/s12879-021-06366-3)
Supplement: Supplementary file 3 — Additional file 3: Figure S2. Calculation of TBR value based on SUV measurements. [file 12879_2021_6366_MOESM3_ESM.pptx]

## Slide 1
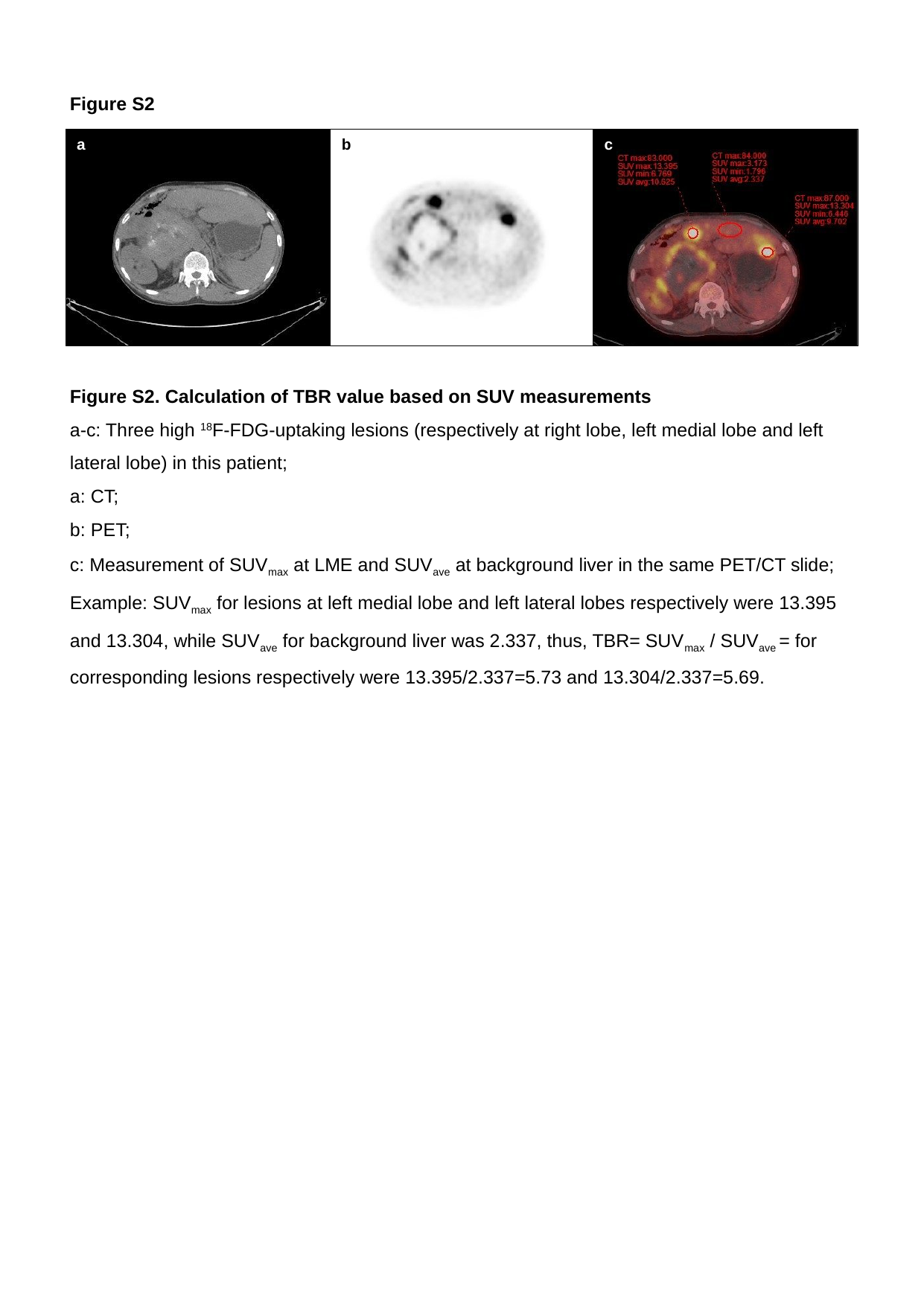

Figure S2
a
b
c
Figure S2. Calculation of TBR value based on SUV measurements
a-c: Three high 18F-FDG-uptaking lesions (respectively at right lobe, left medial lobe and left lateral lobe) in this patient;
a: CT;
b: PET;
c: Measurement of SUVmax at LME and SUVave at background liver in the same PET/CT slide;
Example: SUVmax for lesions at left medial lobe and left lateral lobes respectively were 13.395 and 13.304, while SUVave for background liver was 2.337, thus, TBR= SUVmax / SUVave = for corresponding lesions respectively were 13.395/2.337=5.73 and 13.304/2.337=5.69.
