# Supplementary material for: Quantitative evaluation of range and metabolic activity of hepatic alveolar echinococcosis lesion microenvironment using PET/CT and multi-site sampling method
Source: BMC Infect Dis. 2021 Jul 23;21:702. doi: 10.1186/s12879-021-06366-3 (PMC8299608; doi:10.1186/s12879-021-06366-3)
Supplement: Supplementary file 4 — Additional file 4: Figure S3. Calculation of LME range indicated by PET. [file 12879_2021_6366_MOESM4_ESM.pptx]

## Slide 1
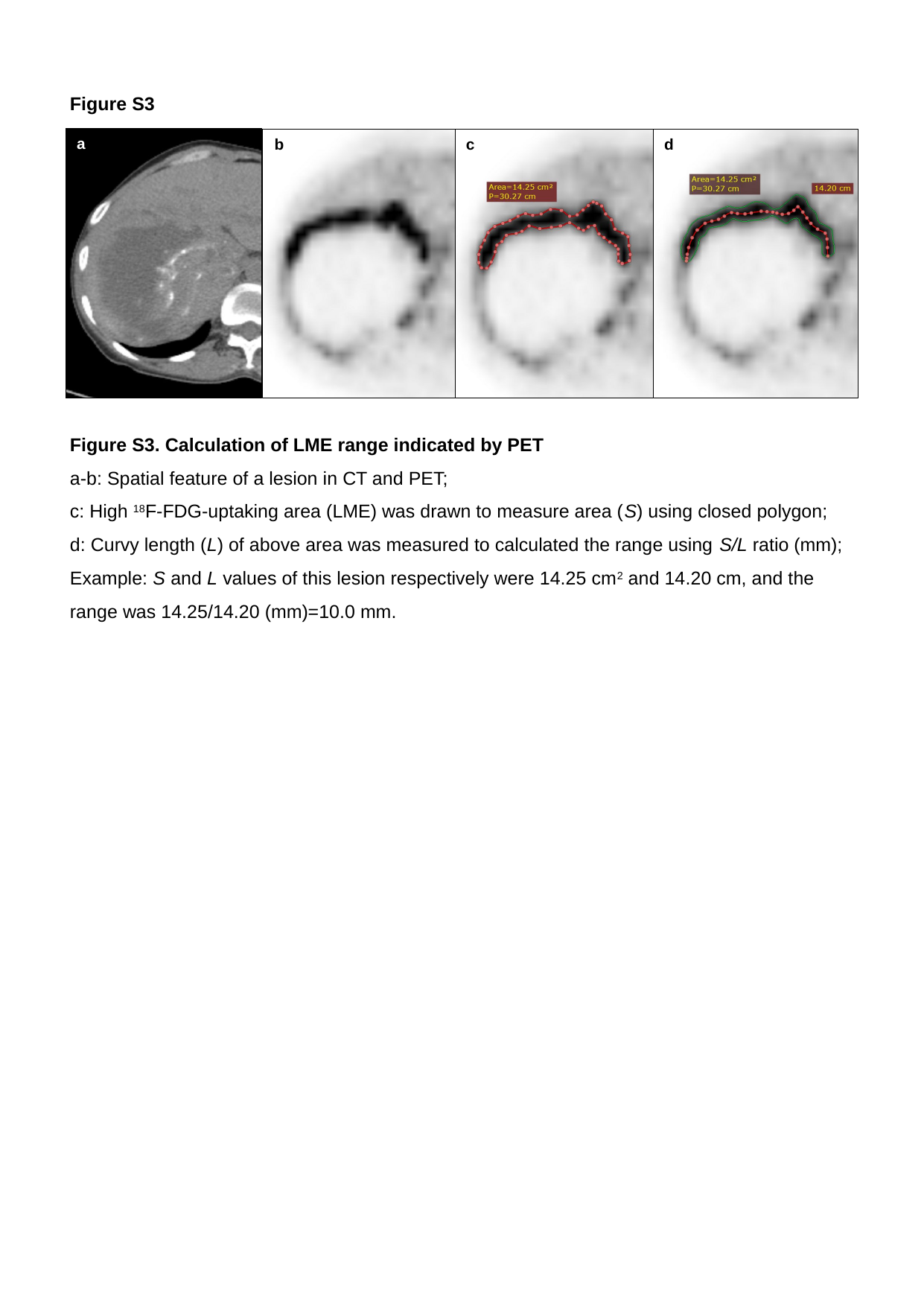

Figure S3
a
b
c
d
Figure S3. Calculation of LME range indicated by PET
a-b: Spatial feature of a lesion in CT and PET;
c: High 18F-FDG-uptaking area (LME) was drawn to measure area (S) using closed polygon;
d: Curvy length (L) of above area was measured to calculated the range using S/L ratio (mm);
Example: S and L values of this lesion respectively were 14.25 cm2 and 14.20 cm, and the range was 14.25/14.20 (mm)=10.0 mm.
