# Supplementary material for: Quantitative evaluation of range and metabolic activity of hepatic alveolar echinococcosis lesion microenvironment using PET/CT and multi-site sampling method
Source: BMC Infect Dis. 2021 Jul 23;21:702. doi: 10.1186/s12879-021-06366-3 (PMC8299608; doi:10.1186/s12879-021-06366-3)
Supplement: Supplementary file 5 — Additional file 5: Figure S4. Representative immune cell infiltration in each groups and quantification of LME range using MSS method. [file 12879_2021_6366_MOESM5_ESM.pptx]

## Slide 1
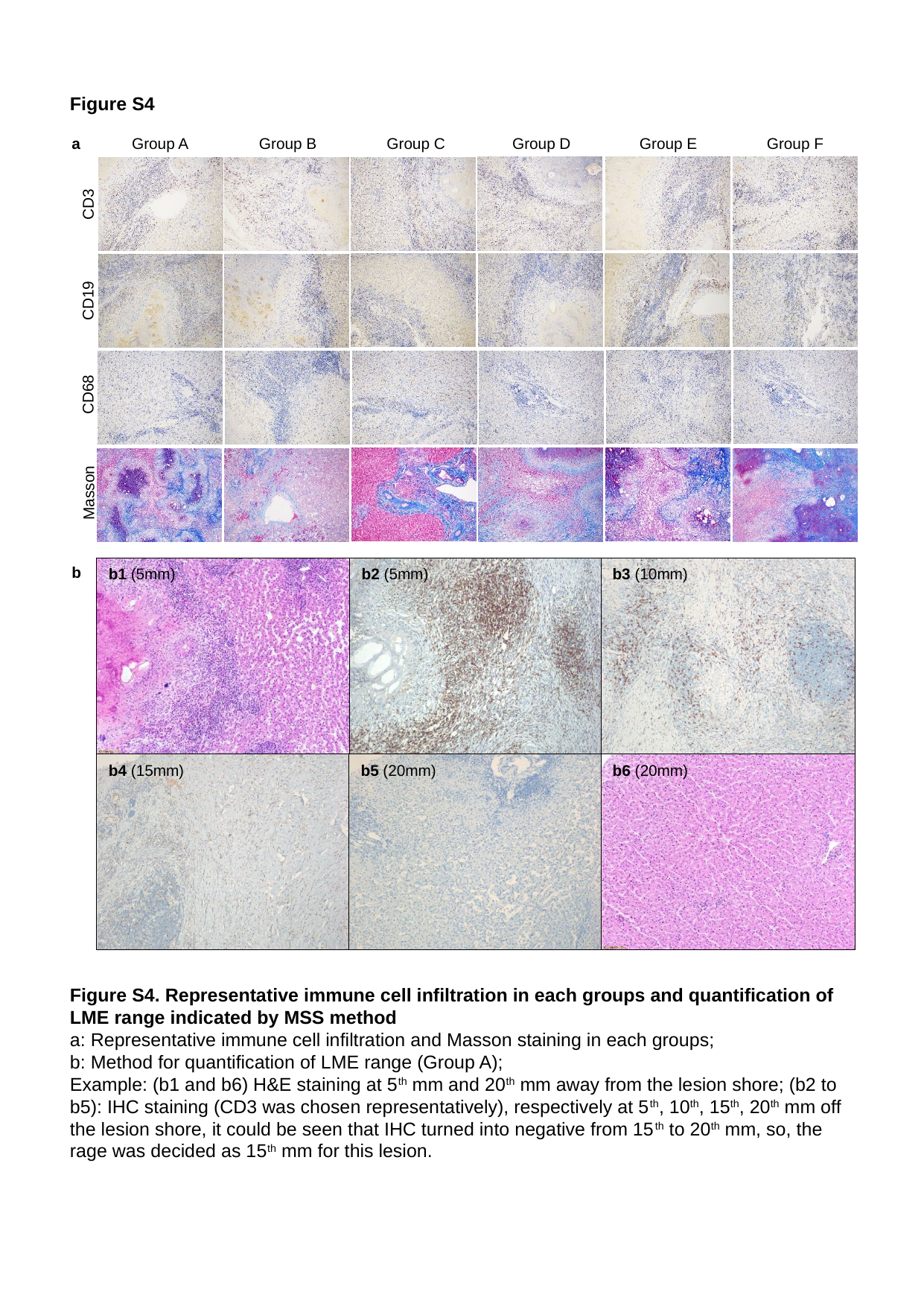

Figure S4
Group E
Group A
Group B
Group C
Group D
Group F
a
CD3
CD19
CD68
Masson
b
b2 (5mm)
b3 (10mm)
b1 (5mm)
b5 (20mm)
b6 (20mm)
b4 (15mm)
Figure S4. Representative immune cell infiltration in each groups and quantification of LME range indicated by MSS method
a: Representative immune cell infiltration and Masson staining in each groups;
b: Method for quantification of LME range (Group A);
Example: (b1 and b6) H&E staining at 5th mm and 20th mm away from the lesion shore; (b2 to b5): IHC staining (CD3 was chosen representatively), respectively at 5th, 10th, 15th, 20th mm off the lesion shore, it could be seen that IHC turned into negative from 15th to 20th mm, so, the rage was decided as 15th mm for this lesion.
