# Supplementary material for: Quantitative evaluation of range and metabolic activity of hepatic alveolar echinococcosis lesion microenvironment using PET/CT and multi-site sampling method
Source: BMC Infect Dis. 2021 Jul 23;21:702. doi: 10.1186/s12879-021-06366-3 (PMC8299608; doi:10.1186/s12879-021-06366-3)
Supplement: Supplementary file 6 — Additional file 6: Figure S5. Distribution of TBR values, PET/CT and MSS indicated LME ranges regarding lesion size. [file 12879_2021_6366_MOESM6_ESM.pptx]

## Slide 1
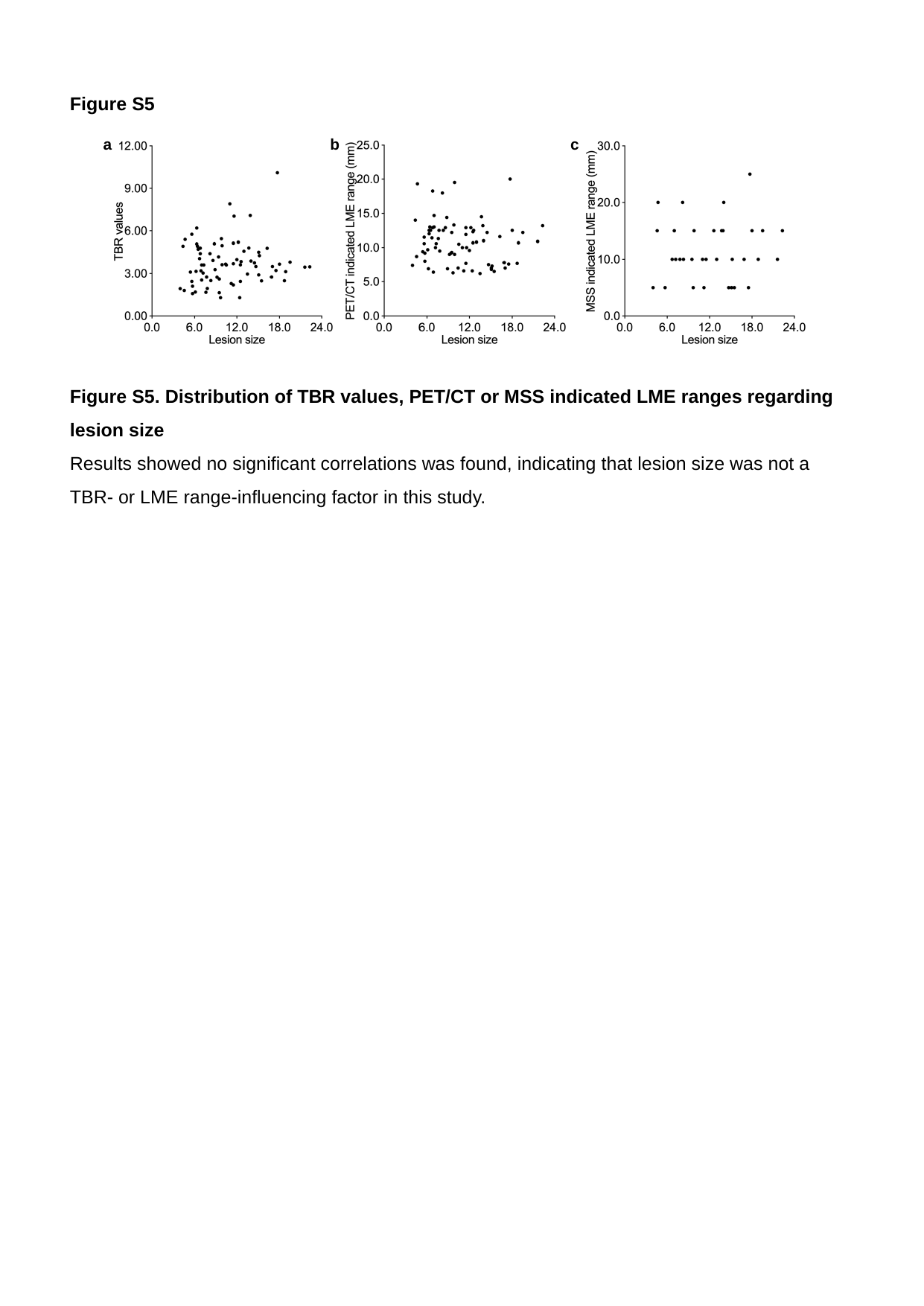

Figure S5
b
c
a
Figure S5. Distribution of TBR values, PET/CT or MSS indicated LME ranges regarding lesion size
Results showed no significant correlations was found, indicating that lesion size was not a TBR- or LME range-influencing factor in this study.
