# Supplementary material for: Quantitative evaluation of range and metabolic activity of hepatic alveolar echinococcosis lesion microenvironment using PET/CT and multi-site sampling method
Source: BMC Infect Dis. 2021 Jul 23;21:702. doi: 10.1186/s12879-021-06366-3 (PMC8299608; doi:10.1186/s12879-021-06366-3)
Supplement: Supplementary file 7 — Additional file 7: Figure S6. Distribution of TBR values, PET/CT and MSS indicated LME ranges regarding different clinical stages. [file 12879_2021_6366_MOESM7_ESM.pptx]

## Slide 1
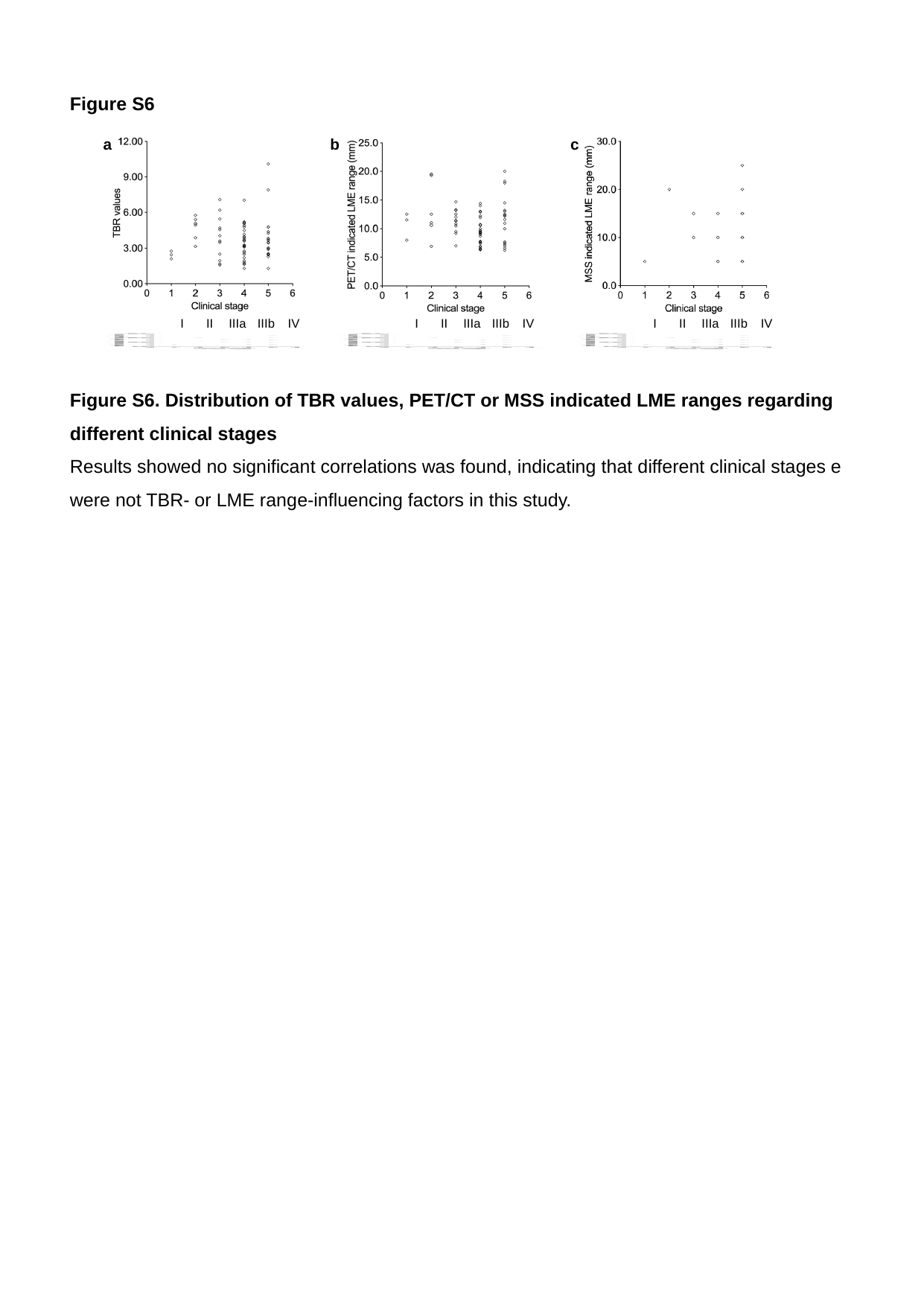

Figure S6
b
c
a
I
II
IIIa
IIIb
I
II
IIIa
IIIb
I
II
IIIa
IIIb
IV
IV
IV
Figure S6. Distribution of TBR values, PET/CT or MSS indicated LME ranges regarding different clinical stages
Results showed no significant correlations was found, indicating that different clinical stages e were not TBR- or LME range-influencing factors in this study.
